# TLD1433-Mediated Photodynamic Therapy with an Optical Surface Applicator in the Treatment of Lung Cancer Cells In Vitro

**DOI:** 10.3390/ph13070137

**Published:** 2020-06-28

**Authors:** Sarah Chamberlain, Houston D. Cole, John Roque, David Bellnier, Sherri A. McFarland, Gal Shafirstein

**Affiliations:** 1 Department of Cell Stress Biology, Photodynamic Therapy Center, Roswell Park Comprehensive Cancer Center, Buffalo, NY 14203, USA; sarah.chamberlain@roswellpark.org (S.C.); david.bellnier@roswellpark.org (D.B.); 2 Department of Chemistry and Biochemistry, The University of Texas at Arlington, Arlington, TX 76019-0065, USA; houston.cole@mavs.uta.edu (H.D.C.); john.roque@uta.edu (J.R.III); 3 Department of Chemistry and Biochemistry, The University of North Carolina at Greensboro, Greensboro, NC 27402-6170, USA

**Keywords:** TLD1433, optical surface applicator, intra-operative photodynamic therapy, PDT, light simulation

## Abstract

Intra-operative photodynamic therapy (IO-PDT) in combination with surgery for the treatment of non-small cell lung cancer and malignant pleural mesothelioma has shown promise in improving overall survival in patients. Here, we developed a PDT platform consisting of a ruthenium-based photosensitizer (TLD1433) activated by an optical surface applicator (OSA) for the management of residual disease. Human lung adenocarcinoma (A549) cell viability was assessed after treatment with TLD1433-mediated PDT illuminated with either 532- or 630-nm light with a micro-lens laser fiber. This TLD1433-mediated PDT induced an EC_50_ of 1.98 μM (J/cm^2^) and 4807 μM (J/cm^2^) for green and red light, respectively. Cells were then treated with 10 µM TLD1433 in a 96-well plate with the OSA using two 2-cm radial diffusers, each transmitted 532 nm light at 50 mW/cm for 278 s. Monte Carlo simulations of the surface light propagation from the OSA computed light fluence (J/cm^2^) and irradiance (mW/cm^2^) distribution. In regions where 100% loss in cell viability was measured, the simulations suggest that >20 J/cm^2^ of 532 nm was delivered. Our studies indicate that TLD1433-mediated PDT with the OSA and light simulations have the potential to become a platform for treatment planning for IO-PDT.

## 1. Introduction

There is no effective therapy for patients with non-small cell lung cancer (NSCLC) with pleural dissemination or malignant pleural mesothelioma [1,2,3]. Intraoperative photodynamic therapy (IO-PDT) with surgery has been reported to improve overall survival in the treatment of NSCLC and malignant mesothelioma, when compared to historical data [4,5,6]. The current IO-PDT regimen involves systemic administration of a photosensitizer (PS) approximately 24–48 h prior to surgery [7]. During surgery, after removal of all macroscopic resectable cancer cells and before closing the surgical wound, a therapeutic laser light is delivered to activate the PS and ablate residual viable cancer cells that could remain in the cavity [8]. The light delivery is accomplished with a handheld light source that the treating physician moves within the thoracic cavity to administer a prescribed threshold light dose (fluence, J/cm^2^) [1,5,7,8]. The delivered light is measured with isotropic detectors that are secured within close-end catheters sutured in about eight anatomical sites that are at high risk for local recurrence due to retention of microscopic cancer cells within the thoracic cavity. 

Here, we report on the potential use of a novel ruthenium (Ru)-based PS (TLD1433) in ablating lung cancer cells. TLD1433 is a Ru(II) polypyridyl complex that can be classified as a metal-organic dyad. It contains a metal center that facilitates efficient population of triplet excited states (either directly or indirectly) and an organic α-terthienyl group that affords a triplet intraligand charge transfer (^3^ILCT) excited state with a prolonged lifetime and high sensitivity to oxygen. The ^3^ILCT states can be populated (i) indirectly via singlet metal-to-ligand charge transfer (^1^MLCT) and ^1^ILCT excited states that are formed following absorption of green light, or (ii) directly with red light. The PDT effects obtained with green light are more potent than those with red light owing to the lower molar extinction coefficients of TLD1433 at the red wavelengths. The attenuated PDT effects at longer wavelengths can be improved by formulating TLD1433 with transferrin (Rutherrin), and this has been demonstrated using bladder cancer cells [9]. The choice of wavelength will depend on the desired tissue penetration depth, with green light being preferred when the penetration depth must be kept minimal (to preserve healthy tissue) and red light when a larger margin of treatment is needed.

TLD1433 is currently being evaluated in a phase II clinical trial for treating high-risk non-muscle invasive bladder cancer (NMIBC) with PDT. The treatment uses green light to avoid damaging the underlying urothelial muscle tissue. In this indication, TLD1433 demonstrates high retention in bladder cancer cells when administered through instillation (in the bladder) 1 h prior to light delivery [10,11,12]. The initial results suggest that this treatment is safe, while inducing effective tumor regression. We suggest that TLD1433-mediated IO-PDT can also be used to treat pleural malignancy. We propose to administer TLD1433 by instillation with sterile saline, in the thoracic cavity. Currently, instillation with sterile saline or intralipid is used to improve optical index matching when administering IO-PDT in the thoracic cavity [7]. We expect that TLD1433 will also exhibit high retention in cancer cells compared to normal lung tissue.

In this paper we propose to activate TLD1433 with surface illumination using a recently developed optical surface applicator (OSA) [13]. The OSA was designed specifically for efficient light delivery in IO-PDT in the thoracic cavity. So far, the OSA has not yet been validated with TLD1433. The purpose of the present study was to test whether the OSA could be used to activate TLD1433 for the destruction of lung cancer cells in vitro before moving to in vivo studies. The OSA’s novel construction allows precise adjustments of the light irradiance (mW/cm^2^) and fluence that are key parameters for effective PDT [14,15]. The OSA includes optical fibers for laser light delivery, and dosimetry fibers for light measurements. A detailed description of the OSA can be found in Chamberlain et al. 2019 [13]. Briefly, the OSA is made of a flexible silicon-based mesh of interconnected spheres 10-mm in diameter with parallel channels that enable placement of optical fibers. The fibers are at fixed distance of 5 mm from the mesh surface. This design is expected to reduce the time of light administration with improved control of light irradiance and fluence [13]. Herein, we also present the first simulation of light irradiance and fluence propagation from the OSA.

This paper is the first report that highlights the potential use of TLD1433-mediated PDT with the OSA in the treatment of human adenocarcinoma (A549) cells. We studied the response of A549 cells to TLD1433 with two clinically approved light wavelengths: 630 and 532 nm. The spatial distribution of cell viability was compared to the distribution of the simulated light irradiance and fluence, laying the foundation for a pretreatment planning for TLD1433-mediated IO-PDT with OSA for pleural malignancy.

## 2. Results

### 2.1. In Vitro PDT with Surface Illumination

**Figure 1:**
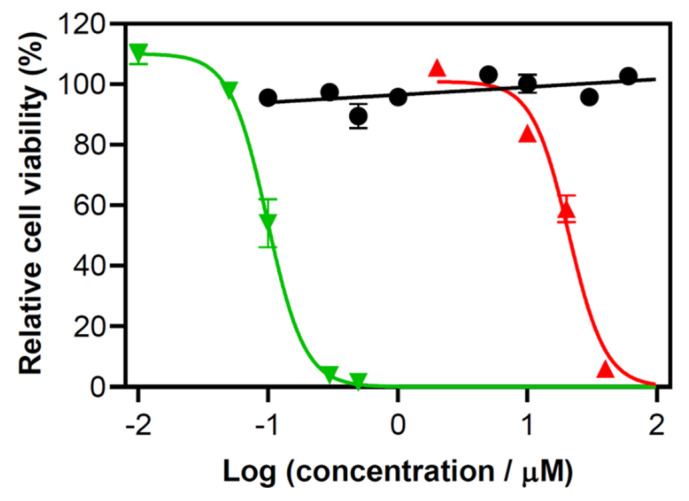
Relative cell viability of human lung adenocarcinoma (A549) cells treated with ruthenium-based photosensitizer (TLD1433). Cell viability was determined by resazurin assay. No exposure to light (black bold circles), irradiation at 532-nm light (green downwards triangle) at 28 mW/cm^2^ for 20 J/cm^2^, and 630-nm light (red upwards triangles) at 65 mW/cm^2^ for 230 J/cm^2^. Error bars represent standard error of the mean. Cells were treated with the following concentrations of TLD1433 before exposure to 532 nm light of 0.01, 0.05, 0.1, 0.3 and 0.5 µM. Similarly, cells were treated with TLD1433 concentrations of 2, 10, 20, and 40 µM before 630 nm light illumination. TLD1433 only (no exposure to light) was assessed at higher concentrations of TLD1433: 0.1, 0.3, 0.5, 1, 5, 10, 30, and 60 µM. In vitro experiments were completed in triplicate.

TLD1433-mediated PDT was evaluated with human lung adenocarcinoma (A549) cells. TLD1433 was nontoxic toward A549 cells over the concentration range investigated in the absence of a light treatment. The effective concentration required to reduce cell viability by 50% (EC_50_) was much greater than the highest concentration tested (60 μM) (Figure 1). Upon irradiation with 532 nm light (28 mW/cm^2^, 20 J/cm^2^), the EC_50_ dropped to 0.099 ± 0.001 μM yielding a phototherapeutic index (ratio of dark to light EC_50_ values) of >>600. The photocytotoxicity was attenuated by more than 200-fold with 630-nm light (65 mW/cm^2^, 230 J/cm^2^), which resulted in an EC50 value of 20.9 ± 3.5 μM (Figure 1). The EC_50_ dose products obtained with green and red light were 1.98 μM·(J/cm^2^) and 4807 μM (J/cm^2^), respectively, resulting in a 2400-fold difference between the two light conditions despite only a 40-fold difference in the number of photons absorbed by TLD1433 at the two different wavelengths. 

**Figure 2:**
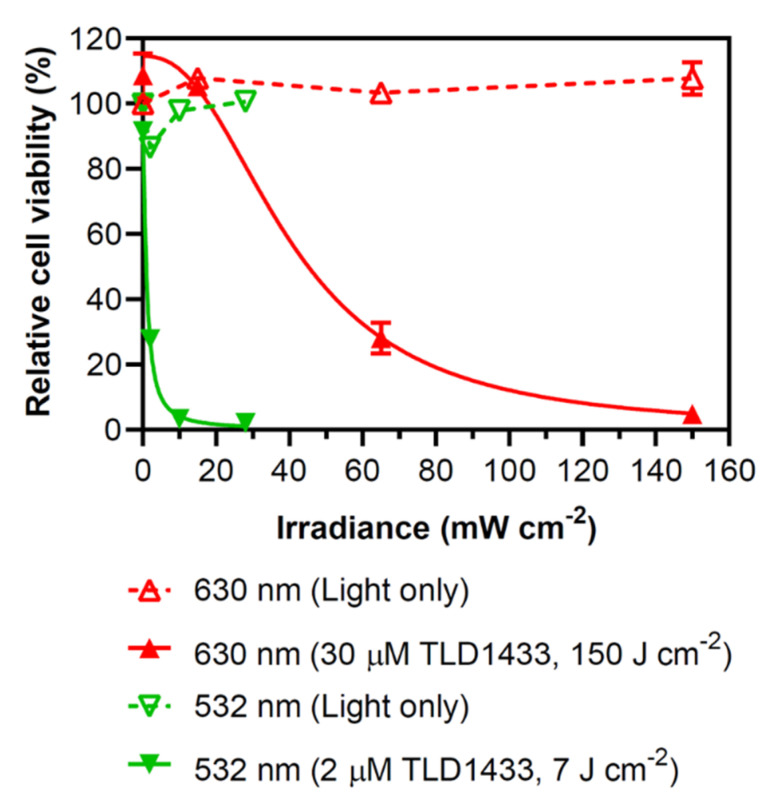
Relative cell viability of A549 cells treated with 630-nm (150 J/cm^2^) or 532-nm (7 J/cm^2^) laser light with and without TLD1433. Cell viability was evaluated with rezasurin cell assay. In vitro experiments were completed in triplicate.

The two light conditions exhibited a different irradiance dependence (Figure 2), and similar photocytotoxicity could be obtained between the wavelengths by changing the concentration of TLD1433, the fluence, and the irradiance. Increasing the irradiance to 150 mW/cm^2^ at 630 nm with 4500 μM (J/cm^2^) induced a similar photocytotoxicity as 532 nm using an irradiance of 28 mW/cm^2^ with 14 μM (J/cm^2^), with no significant toxicity observed when cells were treated with light alone (Figure 2). 

### 2.2. In Vitro PDT with OSA Illumination

**Figure 3:**
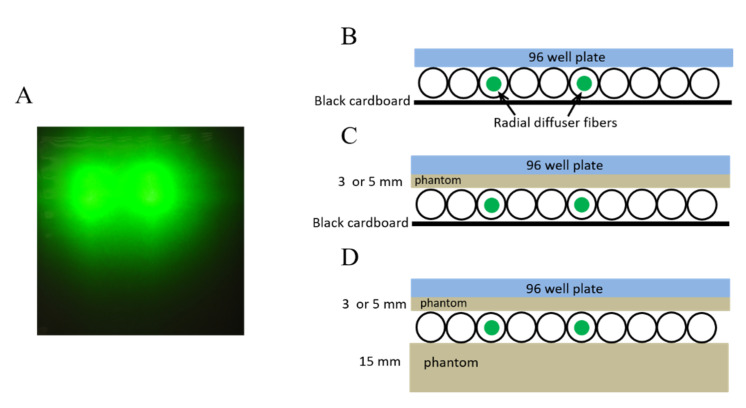
(**A**) A photo of a 3-mm phantom over the optical surface applicator (OSA) with two 2-cm radial diffusers used for the in vitro light administration. Schematic configuration of the OSA, the 96-well plate and phantoms are shown in (**B**) where 96-well plate was placed on top of the OSA silicon beads, (**C**) where 96-well plate was placed on 3- or 5-mm phantoms atop the OSA and (**D**) same as (**C**), where the OSA was placed on a 15-mm phantom (backscatter phantom).

A549 cells, seeded in a 96-well plate, were treated with TLD1433 then illuminated either directly with the OSA or with tissue mimicking phantoms present (Figure 3). Areas of cell viability at the surface of the OSA were similar between plates treated with or without the 15-mm backscatter phantom. After passage through 3- or 5-mm phantoms, cell viability was lower in multiwall plates treated with the 15-mm backscatter phantom versus the plates treated without a backscatter phantom (Figures S1 and S2). The light simulations suggest that an increased irradiance occurred when the 15-mm phantom acting as a backscatter was placed below the OSA. 

**Figure 4:**
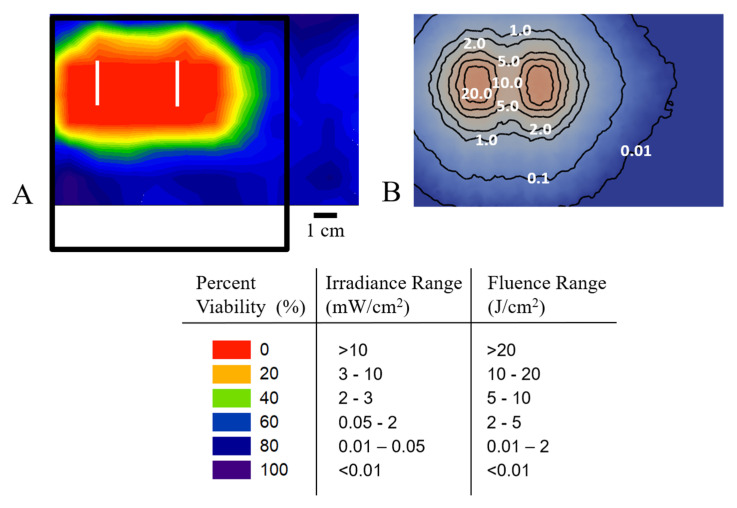
(**A**) Color map of percent cell viability of A549 cells across the 96-well plate. (**B**) The calculated irradiance distribution at the interface of the plate/OSA. The black frame and white lines indicate the OSA and fibers’ position, respectively. The cells were treated with 10 μM TLD1433 and OSA with 532-nm light administration through a 3-mm phantom for 278 s. In vitro experiments were completed in triplicate.

The 3- and 5-mm phantoms atop of the OSA attenuated the light and resulted in higher cell viability when compared to no phantom. However, there was still an effective therapy observed with the 3-mm phantom. In Figure 4, we demonstrate the relationship between cell viability and light irradiance and fluence. The light was delivered through the OSA with a 3-mm phantom on top and the 15-mm backscatter below. Figure 4A depicts a color map of the A549 cell viability, and Figure 4B shows the corresponding calculated irradiance distribution at the surface of the cells (i.e., at the interface of a 3-mm phantom and the bottom of the 96-well plate). The cells were treated with 10 μM TLD1433, and the fluence simulations suggest that 5 J/cm^2^ induced the EC_50_, while 20 J/cm^2^ translates to a 100% loss in cell viability (Figures S1 and S2). The position of the OSA relative to the plate also allows for investigation of cell viability in regions near the OSA. On the rightmost edge of the plate, where the plate was not in direct contact with the OSA (Figure 4A), cell viability remained >60%. This indicates the OSA position and fiber location will allow for controlled light delivery to a region of interest (Figure 4A, Figures S1 and S2). 

## 3. Discussion

We evaluated the response of the aggressive human lung cancer A549 cell line to TLD1433-mediated PDT with both 532- and 630-nm light delivered from a laser fiber with a micro lens. The 532-nm light was found to be more potent than 630-nm light. It has been reported that TLD1433 is a reactive oxygen species generator, with a singlet oxygen quantum yield near unity [9,10]. The photophysical model of TLD1433 suggests that 532-nm light populates excited ^1^MLCT/^1^ILCT states that decay to reactive ^3^ILCT states that sensitize singlet oxygen with high efficiency, whereas 630-nm light populates ^3^MLCT states that are spin forbidden and, once formed, generate singlet oxygen less effectively [10]. The difference in photocytotoxicities elicited by these states at the two different wavelengths could be attributed to an increase in the production of ^1^O_2_ or other reactive oxygen species (ROS), and thus a more effective and potent photoreaction at 532 nm in comparison to 630-nm light, although this is not yet confirmed. At 532 nm, we also observed a steeper increase in potency with irradiance in comparison to 630-nm light (Figure 2). These data suggest that 532 nm is associated with a higher production of ROS in comparison to 630-nm light, when there is sufficient oxygen in vitro. More in vivo work is needed, and underway, to assess this speculation in vivo. 

**Figure 5:**
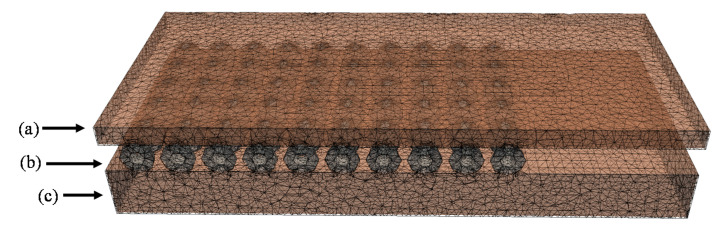
A cross section view of the geometric model and mesh used in the Monte Carlo simulation of light propagation through the OSA (**b**) with a 3- or 5-mm phantom above (**a**). The OSA was placed on a 15-mm phantom (**c**). There were as many as 1.8 × 10^6^ elements in the mesh of the entire geometry.

The 532-nm light, that was found to be potent against the A549 cells, also has a shallow (3–5 mm) tissue penetration depth and thus has the potential to inflict less collateral damage to underlying healthy tissue. We therefore suggest that this treatment is a good candidate for IO-PDT in the thoracic cavity, where underlying sensitive structures need to be protected. We then evaluated the A549 cell response to the OSA with 532-nm light at 3- and 5-mm depth, by using tissue phantoms to mimic depth of penetration. The OSA was found to be effective in delivering 532-nm light irradiance and fluence to induce cell death at 3 mm. The OSA was specifically developed for IO-PDT in the thoracic cavity [13], and its construction makes it possible to develop a pretreatment plan to simulate the fluence and irradiance (Figure 4 and Figure 5). These Monte Carlo (MC) simulations were used to assess the relationship between the calculated irradiance and fluence distribution and cell viability. TLD1433-treated A549 cells growing in a 96-well plate were illuminated with the OSA and assessed for viability. Cell viability was determined over the OSA light field, both at the surface, and after passage through a tissue-mimicking phantom. The results (Figure 4) revealed that the OSA delivered an effective light irradiance and fluence to a planned treatment area where a uniform cell death was measured in the illuminated region. The OSA enabled the delivery of precise and consistent light, confirmed by constructing viability maps at each phantom depth. Our simulations also suggest that the presence of tissue backscatter, which is often the case in areas such as the thoracic cavity, can affect light delivery. This result agrees with the dosimetry measurements performed in our previous studies [13] and supports the notion that the OSA can be used for TLD1433-mediated IO-PDT.

In summary, this study suggests that TLD1433-mediated PDT with the OSA can be used to treat human adenocarcinoma (A549) cells. The use of 532 nm is more potent than 630 nm. The FullMonte is a promising platform to develop a pretreatment planning for TLD1433-mediated IO-PDT with OSA. More work is underway to test our treatment planning and tumor response to TLD1433-mediated IO-PDT in the thoracic cavity of animal models. 

## 4. Materials and Methods 

### 4.1. TLD1433

TLD1433 was prepared as previously described [16,17]. Its structure was confirmed by one- and two-dimensional nuclear magnetic resonance (NMR) spectroscopy and electrospray ionization mass spectrometry (ESI-MS). The purity was determined to be >95% using both high-pressure liquid chromatography (HPLC) and NMR [17]. The UV-visible absorption spectrum was measured (data not shown) and agreed with previously reported optical properties [18]. 

### 4.2. Cell Culture

Human adenocarcinoma cells (A549) were purchased from ATCC (ATCC, Manassas, VA, USA). The cells were cultured in Ham’s F-12K Nutrient Mixture, Kaighn’s Mod. All medium was supplemented with 10% fetal bovine serum (FBS) and 1% penicillin streptomycin. Cells were grown in a humidified incubator at 37 °C with 5% CO_2_. For plating, cells were washed with phosphate-buffered saline (pH 7.4), trypsinized using Ethylenediaminetetraacetic acid (EDTA), and counted in a hemocytometer using 0.4% trypan blue. 

### 4.3. Phantom Construction

Tissue-mimicking phantom construction was previously described by our group [13]. Briefly, gel phantoms were constructed using a combination of intralipid as the scattering agent, India ink as the absorber, and agar powder to make the substrate. The intralipid and India ink concentrations were chosen to produce phantoms with reduced scattering coefficient (µ_s_’) of 7 cm^−1^ and an absorption coefficient (µ_a_) of 0.26 cm^−1^ at 630 nm, and µ_s_’ of 7.05 cm^−1^ and µ_a_ of 0.24 cm^−1^ at 532 nm [13]. The phantoms were 3- or 5-mm thick.

### 4.4. In Vitro PDT

Cells were plated in wells at a seeding density of 2500 cells in 100 µL of media. After incubation for 24 h, the media was replaced with media containing TLD1433 to give final concentrations of 0.01 µM to 60 µM. Following 60 min incubation, the media (containing any TLD1433 not taken up by cells) was replaced with fresh media without TLD1433. Within 3-5 min, the TLD1433-treated cells were exposed to the therapeutic light. This procedure of TLD1433 media replacement follows previous methods described by Kaspler et al. [9].

The cells were illuminated with either a 630-nm or 532-nm light. In one set of experiments, the light was delivered through a laser fiber with a micro-lens (front diffuser, FD1, Medlight SA, Ecublens, Switzerland). A 630-nm diode laser (ML-6500-630, Modulight Inc., Tampere, Finland) was used to illuminate the plate with an irradiance of 65 mW/cm^2^ and fluence of 230 J/cm^2^ with a spot size of 3-cm. A 2-W fiber coupled 532-nm diode laser (LSR532H-2W-FC, CivilLaser, Hangzhou, Zhejiang, China) was used to deliver 20 J/cm^2^ with an irradiance of 28 mW/cm^2^ with a spot size of 5-cm. After light treatment, the plates were returned to the incubator for 24 h. 

In another set of experiments, the cells were illuminated with the OSA, using two 2-cm radial diffusers (RD20, Medlight SA, Ecublens, Switzerland) that were placed 3 cm apart in parallel channels (Figure 3A). Each fiber transmitted 532-nm light at 50 mW/cm for 278 s (a total 200 mW/cm for 55.6 J/cm). Plates were either placed in direct contact with the OSA or on top of a 3-mm to 5-mm phantom (Figure 3B–D). This set up mimics the configuration where the OSA light is delivered in one direction towards the pleural in the chest cavity (Figure 3B,C). In another configuration, the OSA was placed on a phantom that acted as a backscattering layer (Figure 3D) that could be present due to surrounding tissue as we previously reported [13]. All PDT-treated cells were evaluated alongside controls, including cells treated with light but not TLD1433 and cells treated with TLD1433 but not light. Controls for assessing cell growth in the absence of TLD1433 or light were also included. All experiments were conducted in triplicate. 

After light treatment, the plates were returned to the incubator (the 37 °C, 5% CO_2_) for 24 h. Cell viability was then measured with a resazurin cell viability assay. Resazurin was incubated (37 °C, 5% CO_2_) with the cells for 4 h, then measured by quantifying fluorescence (excitation at 570 nm, emission at 585 nm) using a Varian, Cary Eclipse Fluorescence Spectrophotometer plate reader. The cell viability was normalized to growth control. Results of PDT response were plotted using Graphpad Prism, while viability maps were generated using SigmaPlot. 

### 4.5. Treatment Planning

Light simulations were performed using an open source Monte Carlo (MC) software package (FullMonte, https://gitlab.com/FullMonte) [19]. A geometrical model was generated to mimic the in vitro experimental set ups shown in Figure 3. The model includes the OSA with the light sources and phantoms. The FullMonte’s Meshtool was used to generate the tetrahedral mesh (with up to 1.8 × 10^6^ elements) shown in Figure 5. For the Meshtool input parameters, the Cell Radius Edge Ratio, which defines the shape of the elements using the ratio of the circumradius of the tetrahedron and the shortest edge length, was kept at 2.0. In addition, the Smooth parameter, a unitless value used to smooth the surface of the mesh in Meshtool, was between 50–100, in order to keep the complex geometry of the beads in the OSA. Setting Smooth above 200 resulted in loss of beads in the OSA. The largest element size was 2.0 mm.

The light propagation was simulated with 10^7^ photon packets emitted from two 2-cm line sources at 3 cm apart within the OSA. This configuration was identical to the set-up of the in vitro OSA light administration. Simulation results were output in photon packet weight. Irradiance calculated as the output* (Total input power (mW/cm2))/(number of simulated photon packets). Fluence was then acquired by multiplying the irradiance by the total time of light delivery. The 96-well plate was not included in the computation. It was assumed that the 96-well is placed at the surface of the OSA or at 3- or 5-mm of phantoms on top of the OSA surface (as in the configurations shown in Figure 4B–D). 

### 4.6. Patents

G.S. and D.B. are co-inventors of a patent application owned by Roswell Park for the OSA that was licensed to Lumeda Inc. S.A.M. is the inventor of two issued patents (9,345,769 and 9,676,806 B2) for TLD1433 that are licensed to Theralase Technologies, Inc. 

## Supplementary Materials

The following are available online at https://www.mdpi.com/1424-8247/13/7/137/s1, Figure S1: Results for cell viability and simulations without a backscatter phantom, Figure S2: Results for cell viability and simulations with a backscatter phantom.
