# Supplementary material for: TLD1433-Mediated Photodynamic Therapy with an Optical Surface Applicator in the Treatment of Lung Cancer Cells In Vitro
Source: Pharmaceuticals (Basel). 2020 Jun 28;13(7):137. doi: 10.3390/ph13070137 (PMC7407920; doi:10.3390/ph13070137)
Supplement: Supplementary file 1 [file pharmaceuticals-13-00137-s001.pdf]

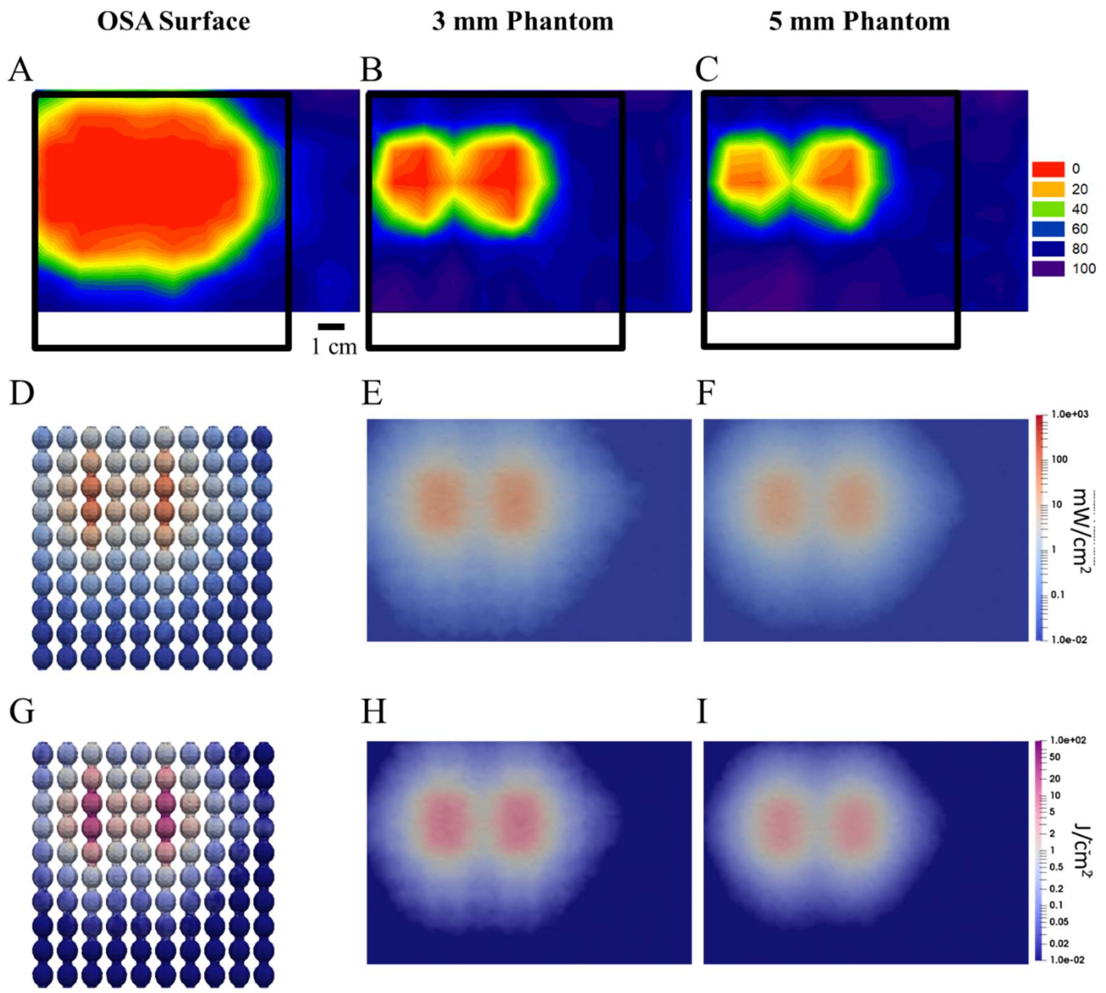

Supplemental Figure S1. Results for cell viability and simulations without a backscatter phantom. (A-C) Color map of percent cell viability of A549 cells across the 96 well plate when treated (A) at the OSA surface, (B) after passage through a 3 mm phantom, and (C) after passage through a 5 mm phantom. (D-F) The calculated irradiance distribution at the interface of the plate and OSA/phantom. (G-I) The calculated fluence distribution at the interface of the plate and OSA/phantom. The black frame and white lines indicate the OSA and fibers' position, respectively. The cells were treated with 10 µM TLD1433 and OSA with 532-nm light administration for 278 seconds.

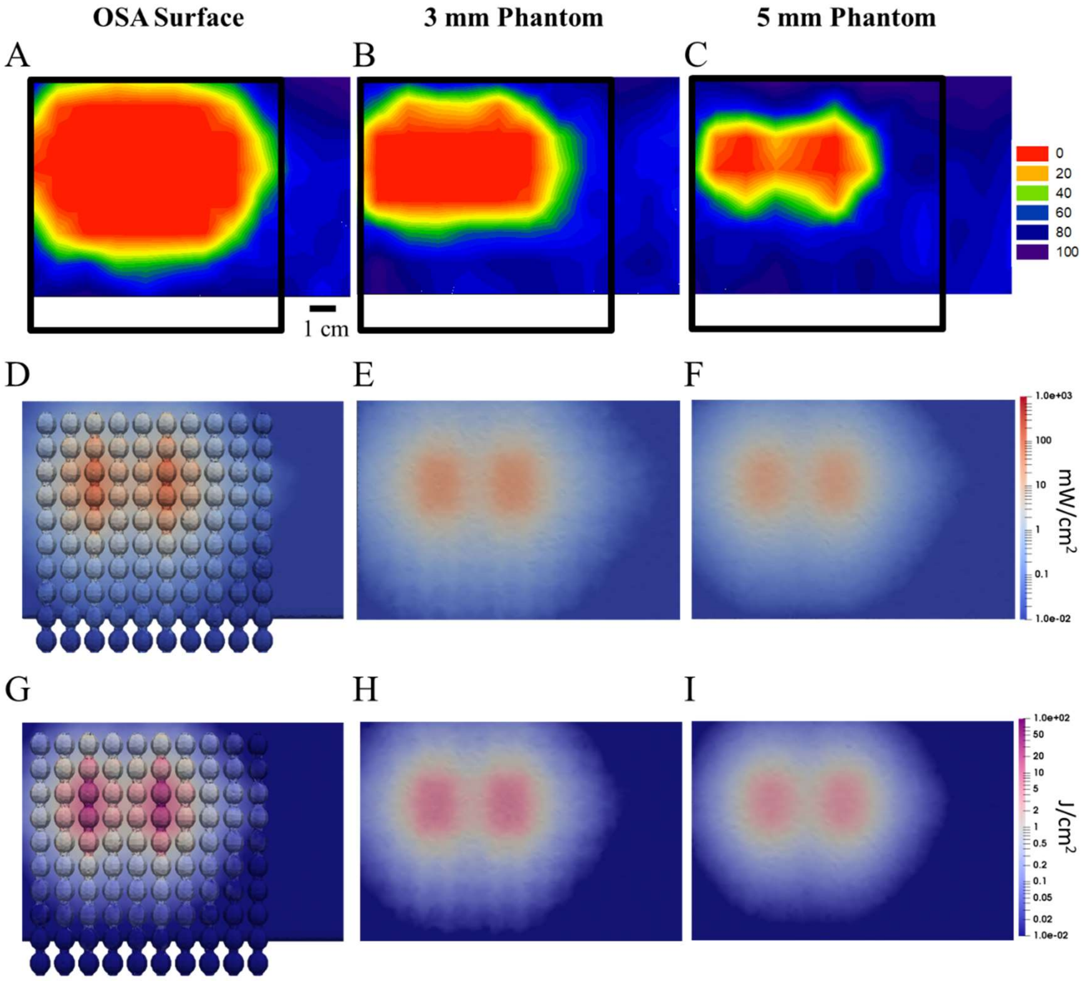

Supplemental Figure S2. Results for cell viability and simulations with a backscatter phantom. (A-C) Color map of percent cell viability of A549 cells across the 96 well plate when treated (A) at the OSA surface, (B) after passage through a 3 mm phantom, and (C) after passage through a 5 mm phantom. (D-F) The calculated irradiance distribution at the interface of the plate and OSA/phantom. (G-I) The calculated fluence distribution at the interface of the plate and OSA/phantom. The black frame and white lines indicate the OSA and fibers' position, respectively. The cells were treated with 10 µM TLD1433 and OSA with 532-nm light administration for 278 seconds.
